# Therapy challenges for NMOSD in a patient with HIV

**DOI:** 10.1177/13524585231199022

**Published:** 2023-09-15

**Authors:** Daniel Engels, Joachim Havla, Stefanie Förderreuther, Tania Kümpfel

**Affiliations:** Institute of Clinical Neuroimmunology, University Hospital, LMU Munich, Munich, Germany; Institute of Clinical Neuroimmunology, University Hospital, LMU Munich, Munich, Germany; Department of Neurology, University Hospital, LMU Munich, Munich, Germany; Institute of Clinical Neuroimmunology, University Hospital, LMU Munich, Munich, Germany

**Keywords:** Neuromyelitis optica (NMO), HIV, rituximab, satralizumab

## Abstract

Neuromyelitis optica spectrum disorder (NMOSD) in people living with HIV (PLWH) is rare and its management can be difficult. Here we report a case of an HIV patient with bilateral vision loss, who was diagnosed with AQP4-IgG-positive NMOSD in 2020 during the COVID-19 pandemic. Rituximab treatment was initiated after attack therapy with corticosteroids and plasma exchange. NMOSD and HIV disease remained stable, but SARS-CoV-2 immune response after repeated vaccinations was insufficient. After switching immunotherapy due to the lack of vaccination response to satralizumab, peripheral B cells reoccurred and a humoral immune response was observed after reapplication of SARS-CoV-2 vaccination. This case illustrates the challenges associated with the treatment of NMOSD in PLWH.

## Introduction

Neuromyelitis optica spectrum disorder (NMOSD) in people living with HIV (PLWH) is a rare condition that has mainly been reported in South Africa.^1^ NMOSD attacks often lead to severe disability, thus prompt initiation of long-term immunotherapy after diagnosis is important. Data on the management of NMOSD in PLWH (NMOSD-PLWH) are limited and can be challenging because immunosuppressive drugs, which are commonly used to treat NMOSD, may worsen HIV disease and harbour the risk of opportunistic infections. Specifically, there is so far no experience with newly approved drugs such as satralizumab for treatment of NMOSD in PLWH. In addition, during the COVID-19 pandemic, immunocompromised individuals, such as patients with NMOSD, who were treated with B cell-depleting drugs, were at an increased risk of severe SARS-CoV-2 infections.^2^ Furthermore, it was shown that SARS-CoV-2 immunization was associated with a poorer humoral immune response while preserving the cellular immune response.^3^ To further complicate matters, COVID-19 morbidity and mortality in PLWH depend on antiviral drug treatment and comorbidities, as well as on CD4^+^ T-cell counts.^4,5^ Ideally, PLWH are fully vaccinated, but their response to SARS-CoV-2 vaccines is expected to be lower, especially in patients with low CD4^+^ T-cell counts.^6^ Here we present an HIV patient who was diagnosed with aquaporin-4 antibody positive (AQP4-IgG+) NMOSD in 2020 and illustrate the treatment challenges in the context of HIV as well as in light of the COVID-19 pandemic.

## Case description

**Figure 1.**
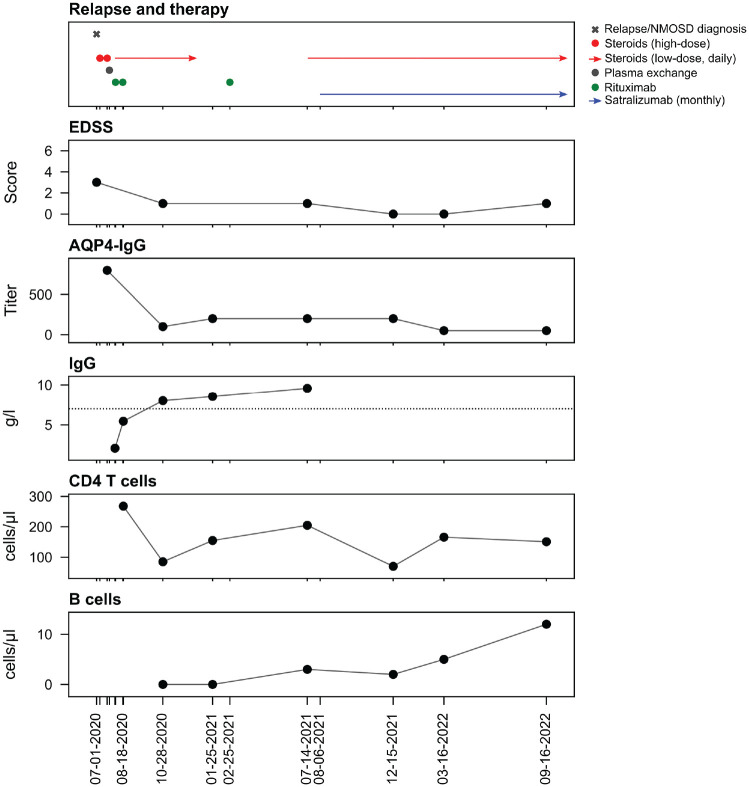
Clinical course, therapy and lab values. Therapy, clinical assessment and laboratory values since the first NMOSD attack. *X*-axis represents the time line of the disease course since the first NMOSD attack (EDSS: Expanded Disability Status Scale, horizontal dotted line indicates lower normal limit).

A 51-year-old patient presented to our ophthalmology department in July 2020 with acute vision loss in the left eye (visual acuity (VA) left: 0.02) and significant scotoma of the right eye. Visual evoked potentials (VEP) were not recordable in the left eye and were delayed in the right eye. Cerebrospinal fluid (CSF) analysis revealed a normal cell count, mild protein elevation (54 mg/dL), and one CSF-specific oligoclonal band. Acute infections were ruled out, and HIV-1-RNA was negative in the serum. Cerebral magnetic resonance imaging (cMRI) showed non-specific white matter lesions while both optic nerves were unremarkable, and spinal cord imaging showed single intramedullary lesions on C5/6, Th2/3 and Th8/9. Optical coherence tomography (OCT) showed no signs of papillitis. Past medical history revealed HIV infection since 2010 (controlled with bictegravir/emtricitabine/tenofovir alafenamide since 2010 and with stable CD4^+^ T-cell counts since many years), hepatitis B virus (HBV) infection in 1994, cured Treponema pallidum infection and anal carcinoma in remission since 2019. After AQP4-IgG in serum was positive (1:800, cell-based assay), AQP4-IgG + NMOSD was diagnosed according to the Wingerchuk criteria (2015). As he did not respond sufficiently to repeated high-dose glucocorticoid therapy, plasma exchange was performed 23 days after disease onset, which led to sustained improvement of vision (VA of both eyes: 1.1, improvement of scotoma). After consultation and thorough discussion with our infectious disease specialist regarding the risks for potential immunosuppressive therapies in the context of HIV, rituximab therapy was initiated (twice 1 g at an interval of 2 weeks) and glucocorticoid therapy was tapered off over several months. Highly active antiretroviral therapy (HAART) was continued unchanged, also considering prophylaxis of rituximab-induced hepatitis B reactivation as an additional effect of tenofovir, and cotrimoxazole added for pneumocystis jirovecii pneumonia (PjP) prophylaxis. The patient as well as his CD4^+^ T-cell counts were closely monitored. NMOSD and HIV remained stable and the patient received a second rituximab cycle (500 mg once) 6 months later. This led to sustained B cell depletion, and serum AQP4-IgG titre decreased to 1:50. CD4 T-cell counts decreased transiently after starting low-dose steroid therapy. In 2021, after the introduction of SARS-CoV-2 vaccines, the patient was vaccinated four times (twice mRNA BNT162b2 BioNTech/Pfizer, Ad26.COV2.S, Janssen, mRNA-1273, Moderna), but did not show a sufficient humoral immune response measured by SARS-CoV-2 spike antibody titre. At that time, severe SARS-CoV-2 infections under treatment with rituximab were reported and cessation of rituximab therapy was discussed for immunosuppressed patients with an insufficient humoral immune response to vaccination. Isolation was not consistently feasible for our patient due to professional reasons. Therefore, after approval of satralizumab in June 2021 in Germany, we decided to switch therapy from rituximab to satralizumab in August 2021. Low-dose oral steroid therapy was added for 3 months in order to avoid attacks during the switching period. Subsequently, the blood B cell count increased, serum AQP4-IgG titre remained stable at low levels (last follow-up March 2023 1:50), and SARS-CoV-2 spike antibodies were detected in the serum after repeated SARS-CoV-2 vaccination. Likewise, both NMOSD and HIV infection were well controlled with satralizumab in combination with HAART up to a 1.5-year follow-up (Figure 1).

## Discussion

Here, we report therapy management in an HIV-NMOSD patient in the context of the COVID-19 pandemic. To date, there is no evidence of an increased occurrence of NMOSD in PLWH. However, there might be a relevant interaction between these comorbidities regarding NMOSD immunotherapy and control of HIV. In general, NMOSD immunotherapy can lower B-cell as well as T-cell counts and increase the risk of infections. Likewise, HIV-associated infections can occur because of low CD4^+^ T-cell counts.

Thus, in PLWH, who require immunosuppression due to autoimmune comorbidities like NMOSD, risk management of infections is crucial. Rituximab, which has been shown to effectively prevent attacks in NMOSD, is used for the treatment of HIV-related B cell lymphoma; however, there is an increased risk of infections, especially for patients with low CD4^+^ T-cell counts.^7^ In addition, the humoral immune response to vaccinations is reduced in patients receiving rituximab, which is a considerable aspect of therapeutic management in the context of the COVID-19 pandemic.^3^ Besides B cell depletion, HIV infection and low CD4 cell counts can be considered as additional risk factors that diminish vaccination efficacy. However, a sufficient vaccine response has been shown in PLWH, especially if HIV infection is controlled by HAART and with CD4 counts of ⩾200 cells/mm^3^.^8^ Recent approvals of interleukin-6- (IL-6-) receptor and complement-targeting NMOSD therapies (satralizumab and eculizumab/ravulizumab, respectively) have expanded therapeutic options for patients with AQP4-IgG+ positive NMOSD. It has been shown that the humoral COVID-19 vaccine response in NMOSD patients treated with anti-IL-6 receptor therapies is higher compared with those treated with anti-CD20 therapies.^9^ Here, we showed that after switching therapy from rituximab to satralizumab in an HIV-NMOSD patient with sufficient HAART, both the HIV and NMOSD disease courses remained stable within a 1.5-year observation period.

## Conclusion

Newly diagnosed NMOSD in patients with HIV infection is rare. This case illustrates the challenges for therapy not only during the pandemic but also underscores the importance of interdisciplinary collaboration. Furthermore, IL-6-receptor-targeted antibodies such as satralizumab could be a novel, safe and effective treatment strategy in PLWH with NMOSD who are under sufficient HAART.
